# Poster Session I- A42 ANTIBIOTICS AND *C. DIFFICILE* INFECTION DIFFERENTIALLY REGULATE MOUSE INTESTINAL TUFT CELL POPULATIONS

**DOI:** 10.1093/jcag/gwaf042.042

**Published:** 2026-02-13

**Authors:** N Al-Emadi, T Steiner

**Affiliations:** Microbiology & Immunology, The University of British Columbia, Vancouver, BC, Canada; Microbiology & Immunology, The University of British Columbia, Vancouver, BC, Canada

## Abstract

**Background:**

Type 2 immune responses play a protective role in mitigating the severity of *Clostridioides difficile* infection (CDI). Specifically, the alarmin cytokine IL-25 has been shown to be protective in murine models. In the intestinal epithelium, IL-25 is primarily produced by tuft cells, specialized chemosensory epithelial cells. Despite the protective role of their key effector cytokine, the dynamics of the tuft cell population during antibiotic-induced dysbiosis and subsequent CDI are poorly characterized.

**Aims:**

This study tested our hypothesis that intestinal tuft cells are depleted following antibiotic treatment and subsequent CDI and aimed to characterize these dynamic changes across different regions of the murine gut.

**Methods:**

C57BL/6 mice (sex-matched, 9-12 weeks) were pre-treated for three days with an antibiotic cocktail dissolved in gel food, followed by intraperitoneal clindamycin injection. Mice were subsequently challenged via oral gavage with 10^5^ CFU of *C. difficile* (VPI 10463) spores. Control groups included untreated mice and antibiotic-treated mice without infection. At day 3 post-infection, distal ileum, cecum, proximal colon, and distal colon tissues were collected and stained for DCLK1 via immunofluorescence. Tuft cell counts were quantified per mm^2^ of intestinal tissue.

**Results:**

Antibiotic treatment and subsequent CDI resulted in distinct, region-specific alterations to intestinal tuft cell populations. In the large intestines, CDI challenge led to a significant reduction in tuft cell density across all tested regions (cecum, proximal colon, distal colon) compared to untreated controls. Antibiotic treatment alone also significantly reduced tuft cell numbers in the cecum and distal colon. In contrast, antibiotic treatment induced a significant tuft cell expansion in the ileum, which was abrogated upon CDI challenge.

**Conclusions:**

Our results reveal that intestinal tuft cell populations are dynamically and differentially regulated by antibiotic treatment and subsequent CDI, leading to a pronounced depletion through the large intestine during infection. The decline of a key immunomodulatory cell type may worsen CDI by impairing type 2 immune responses. Further studies are underway to explore the functional consequences of these cellular changes.

**Funding Agencies:**

Supported by unrestricted research funds

